# Inhibition of the TRPM2 and TRPV1 Channels through *Hypericum perforatum* in Sciatic Nerve Injury-induced Rats Demonstrates their Key Role in Apoptosis and Mitochondrial Oxidative Stress of Sciatic Nerve and Dorsal Root Ganglion

**DOI:** 10.3389/fphys.2017.00335

**Published:** 2017-05-31

**Authors:** Fuat Uslusoy, Mustafa Nazıroğlu, Bilal Çiğ

**Affiliations:** ^1^Department of Plastic Reconstructive and Aesthetic Surgery, Faculty of Medicine, Suleyman Demirel UniversityIsparta, Turkey; ^2^Neuroscience Research Center, Suleyman Demirel UniversityIsparta, Turkey; ^3^Department of Biophysics, Faculty of Medicine, Suleyman Demirel UniversityIsparta, Turkey; ^4^Department of Neuroscience, Institute of Health Sciences, Suleyman Demirel UniversityIsparta, Turkey

**Keywords:** apoptosis, *Hypericum perforatum*, sciatic nerve injury, mitochondrial oxidative stress, TRPM2, TRPV1

## Abstract

Sciatic nerve injury (SNI) results in neuropathic pain, which is characterized by the excessive Ca^2+^ entry, reactive oxygen species (ROS) and apoptosis processes although involvement of antioxidant *Hypericum perforatum* (HP) through TRPM2 and TRPV1 activation has not been clarified on the processes in SNI-induced rat, yet. We investigated the protective property of HP on the processes in the sciatic nerve and dorsal root ganglion neuron (DRGN) of SNI-induced rats. The rats were divided into five groups as control, sham, sham+HP, SNI, and SNI+HP. The HP groups received 30 mg/kg HP for 4 weeks after SNI induction. TRPM2 and TRPV1 channels were activated in the neurons by ADP-ribose or cumene peroxide and capsaicin, respectively. The SNI-induced TRPM2 and TRPV1 currents and intracellular free Ca^2+^ and ROS concentrations were reduced by HP, N-(p-amylcinnamoyl) anthranilic acid (ACA), and capsazepine (CapZ). SNI-induced increase in apoptosis and mitochondrial depolarization in sciatic nerve and DRGN of SNI group were decreased by HP, ACA, and CapZ treatments. PARP-1, caspase 3 and 9 expressions in the sciatic nerve, DRGN, skin, and musculus piriformis of SNI group were also attenuated by HP treatment. In conclusion, increase of mitochondrial ROS, apoptosis, and Ca^2+^ entry through inhibition of TRPM2 and TRPV1 in the sciatic nerve and DRGN neurons were decreased by HP treatment. The results may be relevant to the etiology and treatment of SNI by HP.

## Introduction

Calcium ion (Ca^2+^) is an important messenger in neurons of the body (Kumar et al., 2014). Many physiological functions such as muscle metabolism, neuronal recovery, mitochondrial-reactive oxygen species (ROS) production, and apoptosis were regulated by the intracellular free Ca^2+^ ([Ca^2+^]_i_) concentration (Nazıroğlu, 2007; Kumar et al., 2014). The Ca^2+^ passes the cell membrane with several channels such as chemical gated and voltage gated calcium channel (VGCC). Apart from the well-known channels, transient receptor potential (TRP) cation channel family was recently discovered in different cells. Some subfamilies of the TRP family, such as TRP melastatin 2 (TRPM2) and TRP vanilloid 1 (TRPV1) are activated by oxidative stress (Tominaga and Tominaga, 2005; Nazıroğlu, 2011, 2015). The activator of TRPM2 channel was firstly discovered through activation of adenosine diphosphate ribose (ADPR) pyrophosphatase enzyme of C-terminal tail in the Nudix box motif of the channel by intracellular ADPR (Perraud et al., 2001) and extracellular H_2_O_2_(Hara et al., 2002). Then, ADPR-independent activation mechanism of TRPM2 channel was indicated in a cell line by a single channel patch-clamp study (Nazıroğlu and Lückhoff, 2008). Activation of TRPV1 channel is firstly indicated in dorsal root ganglion neuron (DRGN) by capsaicin (Caterina et al., 2000). Then, involvement of oxidative stress on activation of the TRPV1 channel through activation of NADPH oxidase pathway was indicated by a cell line study (Susankova et al., 2006) and DRGN (Ding et al., 2016) studies. TRPM2 and TRPV1 expression levels are high in the DRGNs and overload Ca^2+^ entry through the channels involved in neuropathic pain (Isami et al., 2013; Pecze et al., 2013; Akpınar et al., 2016) and apoptotic (Hara et al., 2002) processes. The association between overload Ca^2+^ entry through TRPM2 and TRPV1 and peripheral pain intensity has been reported in sciatic nerve injury (SNI)-induced rats (Dolu et al., 2016). Accumulating evidence indicated that importance of oxidative stress, the TRPM2 and TRPV1 channels in DRGN and sciatic nerve injuries has been increasing in experimental animal and human (Facer et al., 2007; Frederick et al., 2007; Haraguchi et al., 2012). On the subject, it was reported that expression levels of TRPM2 and TRPV1 are increased in sciatic nerve and DRGN by spinal cord injury (SCI) and SNI (Frederick et al., 2007; Szigeti et al., 2012; Matsumoto et al., 2016).

It was demonstrated that inflammatory, mechanical injury, and ischemia induces excessive production of ROS, Ca^2+^ entry, and apoptosis through VGCC in neurodegenerative diseases such as SCI and SNI (Fisunov et al., 2000). Association between overload Ca^2+^ entry and excessive production of ROS has also been well-known in neurodegenerative disease (Graphical abstract). Involvement of excessive ROS production and overload Ca^2+^ entry has been emphasized in the generation of neuropathic pain after traumatic injuries (Genovese et al., 2006; Özdemir et al., 2016). Based on these findings, some dietary antioxidants have been tested for their clinical efficacy in treating oxidative stress, apoptosis, and Ca^2+^ entry because they acted to be safe and well-tolerated (Genovese et al., 2006; Alipour et al., 2014; Özdemir et al., 2016). *Hypericum perforatum* (HP) is also known as St John's worth which has been used as a popular plant medicine for treatment of several diseases such as skin wounds, burns, and mental depression (Stojanović et al., 2013). Antioxidant and ROS scavenger effects of flavonoids are well-known for a long time and the main component of HP is flavonoids such as hyperforin, pseudohyperforin, rutin, quercetin, and quercitrin (Kusari et al., 2009; Stojanović et al., 2013). A protective effect of HP on sciatic nerve in rats was recently reported (Mohammadi et al., 2012). Antioxidant and ROS scavenger effects of HP both in DRGN of SCI-induced rats (Uchida et al., 2008; Nazıroğlu et al., 2014a) and neutrophil of patients with inflammatory diseases (Nazıroğlu et al., 2014b,c) were reported. Recently, we observed modulator role of HP on apoptotic, inflammatory and oxidative stress values in muscle, blood and brain of SNI-induced rats (Uslusoy et al., 2017). Therefore, HP may attenuate oxidative stress, apoptosis and Ca^2+^ entry through modulation of TRPM2 and TRPV1 in DRGN and sciatic neurons of SNI-induced rats.

**Graphical Abstract:**
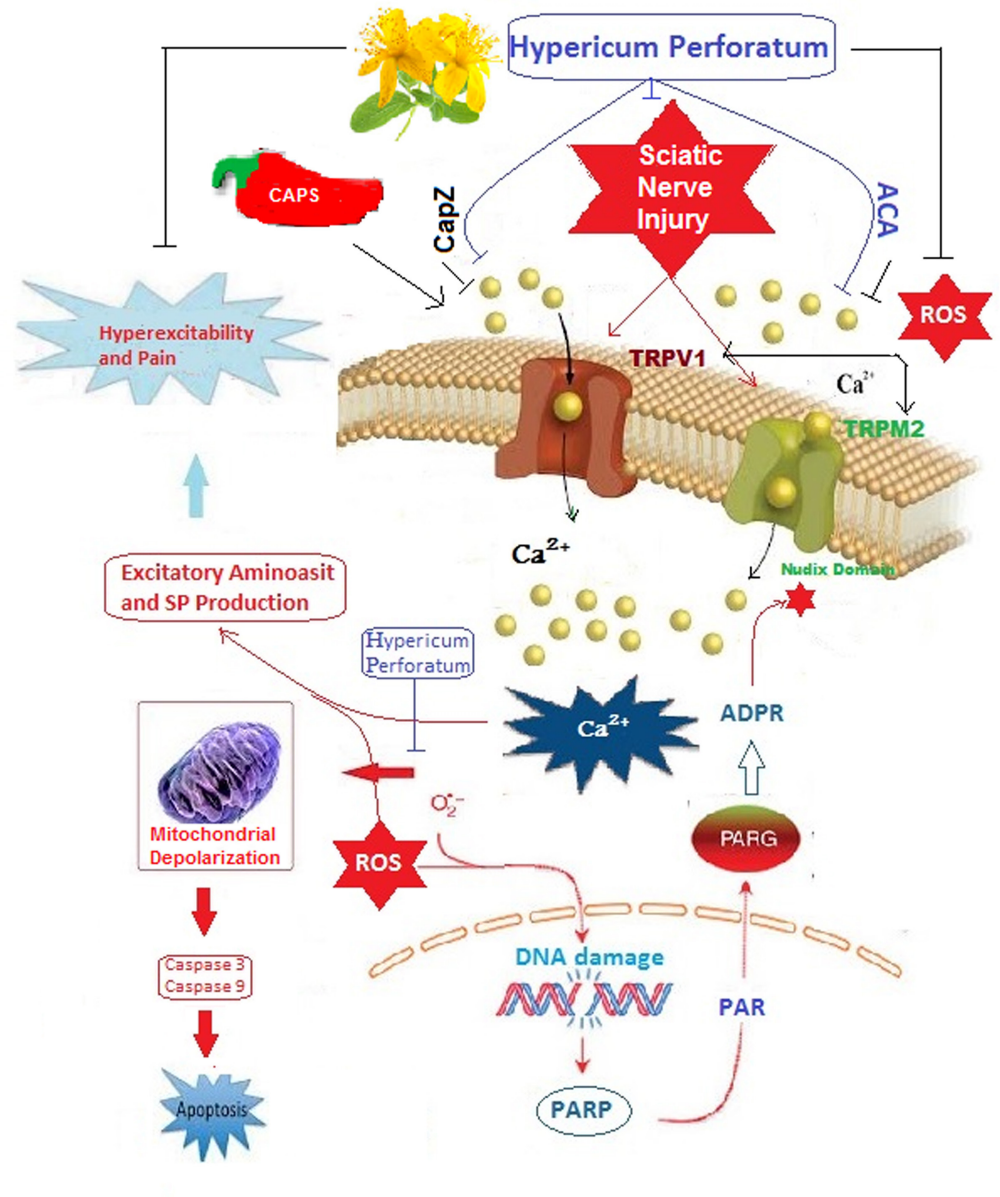
**Poly (ADP-ribose) polymerase (PARP) catalyzes the transfer of ADP-ribose (ADPR) in nucleus during the DNA repair processes**. TRPM2 channel is gated by ADPR and reactive oxygen species (ROS) through activation of ADP-ribose (ADPR) pyrophosphate enzyme in its nudix domain motif although it was inhibited by N-(p-amylcinnamoyl)anthranilic acid (ACA). TRPV1 channel is also activated by ROS and capsaicin (CAPS) but it is inhibited by capsazepine (CapZ).

SNI-induced apoptosis and oxidative stress may be reduced by [Ca^2+^]_i_ concentration through modulation of TRPM2 and TRPV1 channels by HP. To our knowledge, there is no report of HP on apoptosis, oxidative stress and Ca^2+^ entry in SNI-induced rats. The aim of the current study is to determine the molecular mechanism of HP on apoptosis, oxidative stress and Ca^2+^ entry through TRPV1 and TRPM2 regulation in the sciatic nerve and DRGN after SNI.

## Materials and methods

### Animal

We used 40 female Wistar rats (aged between 3–4 months old) in the current study. The animals were housed two per cage, under controlled conditions of room temperature (22°C) and humidity (65–70%), on a 12 h light-dark cycle and allowed free access to commercial feed and tap water. Accessing the feed of the operated animals was facilitated to the rats though using specific cage apparatus in sham and SNI-induced groups for recovery days (3 days) of the operations.

### *Hypericum perforatum* (HP) extract

The HP extract was purchased from Indena (Indena Industria Derivati Naturali) S.p.A. Viale Ortles, Milan, Italy. The extract was mainly containing 0.10–0.30% total hypericin, 6.0% flavonoids, and 6.0% hyperforin (Özdemir et al., 2016; Uslusoy et al., 2017).

### Study groups

**Figure 1:**
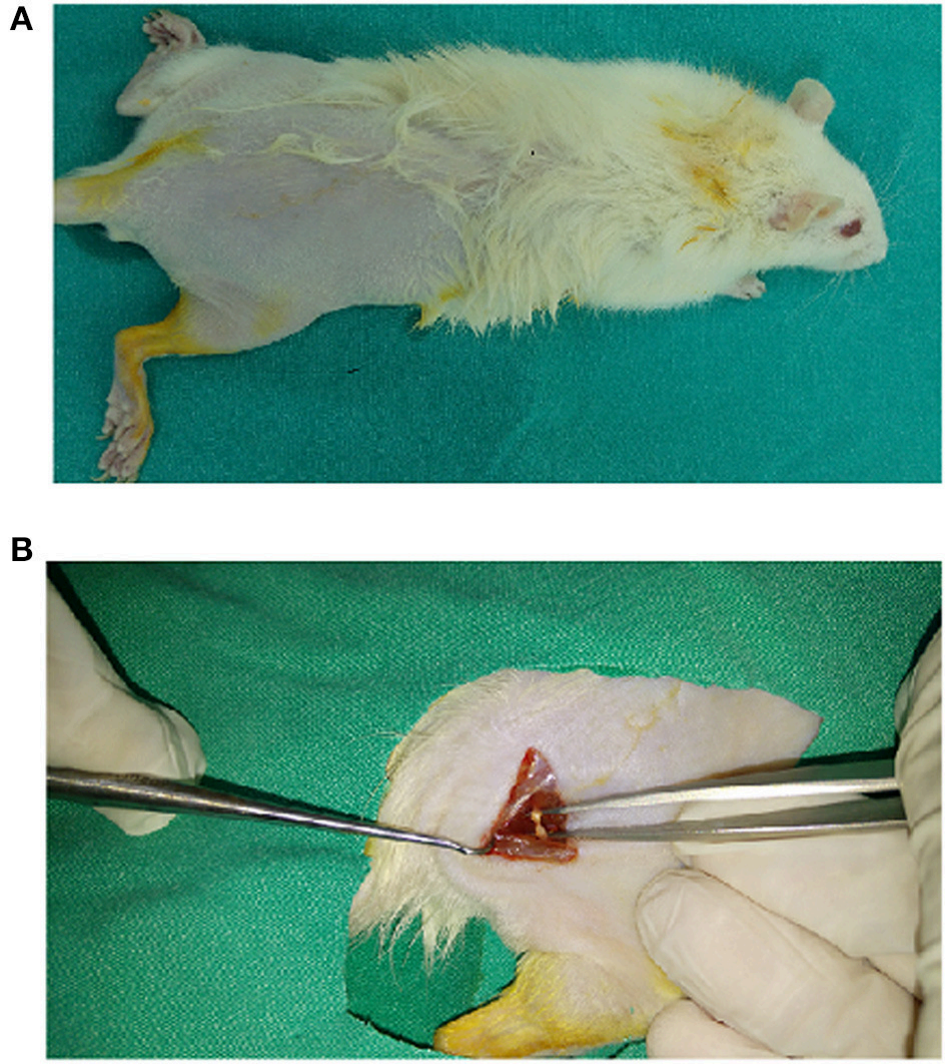
**Induction of sciatic nerve injury in right leg of the rats**. After anesthesia **(A)**, the common sciatic nerve of the right hind paw was exposed the middle of thigh by blunt dissection through the biceps femoris **(B)**. For sciatic nerve crush, a hemostatic sterile clamp was used. The sciatic nerve was crushed for a total of 30 s. Then, the wound was closed with a 2.0 suture and rats were allowed to recover in the postoperative room. In sham-operated rats, the same surgical procedure was followed, the connective tissue was freed, and no ligatures were applied.

The rats were equally divided into five groups (*n* = 8) as follows: The control group had no SNI and treatment. They received one ml of 0.9% w/v saline solution via gastric gavage for 4 weeks. In the sham group, they exposed the same surgical procedure of SNI group, but no ligatures were applied to right leg (Figure 1). In sham+HP group, exposed same procedure of sham group, but the rats were supplemented HP. In the SNI group, they exposed the same surgical procedure of SNI group and ligatures were also applied to right leg. In SNI+HP group, exposed same procedure of SNI group, but the rats were supplemented HP. The HP (30 mg/kg/day) was dissolved in ml of 0.9% w/v saline and it was administrated to the rats via gastric gavage for 4 weeks (Özdemir et al., 2016; Uslusoy et al., 2017). SNI in the SNI group was induced in the rats according to method of Bennett and Xie (1988). In the SNI+HP group, the rats received oral HP (30 mg/kg/day). In the SNI+HP, the rats received HP (same as the sham+HP group) after SNI induction (same as the SNI group).

Twelve hours after the last HP dose administration, all rats were decapitated in accordance with Suleyman Demirel University (SDU) experimental animal legislation. The skin, muscle (Musculus piriformis), sciatic nerve, and DRGN samples were isolated as described in a previous study (Özdemir et al., 2016). In patch-clamp experiment and [Ca^2+^]_i_ concentration assays, the DRGNs were further treated with cumene hyroperoxide (CPx) (0.1 mM) or ADPR (1 mM) and capsaicin (0.01 mM) for activation of TRPM2 and TRPV1 channels, respectively and they were also inhibited the TRPM2 channels blockers, N-(p-amylcinnamoyl) anthranilic acid (ACA and 0.025 mM) and TRPV1 blocker, capsazepine (CapZ and 0.1 mM).

### Induction of SNI and preparation of sciatic nerve and DRGNs

Briefly, the rats were anesthetized by cocktail of xylazine (12.5 mg/kg) and ketamine (100 mg/kg) via intraperitoneally and the common sciatic nerve of the right hind paw was exposed the middle of thigh by blunt dissection through the biceps femoris. For sciatic nerve crush, a hemostatic sterile clamp was used. The sciatic nerve was crushed for a total of 30 s. Then, the wound was closed with a 2.0 suture and rats were exposed to recover in the postoperative room. For excusing the effects of anesthetics and surgical operation on the investigated values, we induced sham group. In sham-operated rats, the same surgical procedure was followed, the connective tissue was freed, and no ligatures were applied. After 3 days of the surgical operation, all animals received gentamicin (5 mg/kg, i.p.) to prevent sepsis.

The DRGN (T13-L5) were carefully dissected from peripheral nerve roots (Nazıroğlu et al., 2014a). The neurons were incubated in DMEM with 1% penicillin-streptomycin in 500 ml of DMEM. The connective tissue was removed and ganglia were treated with collagenase IV (0.28 ml in DMEM), and tyripsin (25,000 units/ml in DMEM for 45 min at 37°C and in an atmosphere containing 95% air and 5% CO_2_. After dissociation with a sterile syringe, the DRGN suspension of medium and high size was obtained by centrifuged at 1,500 g and the medium and high size neurons were removed for the analysis (Akpınar et al., 2016).

### Measurement of [Ca^2+^]_i_ concentration in sciatic nerve and DRGN

In [Ca^2+^]_i_ measurement, extracellular buffer was contained 140 mM NaCl, 5 mM KCl, 1 mM MgCl_2_, 2 mM CaCl_2_, 10 mM 4-(2-hydroxyethyl)-1- piperazineethanesulfonic acid (HEPES), and 5 mM glucose (pH 7.4). Lysis buffer (pH 7.5) contained 20 mM Tris X-100, 150 mM NaCl, 1 mM ethylenediaminetetraacetic acid (EDTA), 1 mM EGTA, 0.1% Triton X-100, 2.5 mM sodium pyrophosphate.

The sciatic nerve and DRGNs (10^6^/ml) were allowed to recover in RPMI-1640 medium for 1 h before being loaded with 2 mM fura-2-AM for 30 min in a water-jacketed cuvette (37°C) with continuous magnetic stirring (Espino et al., 2010). Fluorescence was monitored with a Carry Eclipsys (Inc, Sydney, Australia) spectrofluorometer immediately after 0.1 ml cell suspension was added to 0.9 ml Ca^2+^-containing extracellular medium, by recording excitation signals at 340 and 380 nm and emission signal at 505 nm at 1 s intervals. For calibration of [Ca^2+^]_i_, maximum, and minimum fluorescence values were obtained by adding the detergent Triton X-100 (0.1%) and the Ca^2+^ chelator EGTA (10 mM) sequentially at the end of each experiment. Calculation of the [Ca^2+^]_i_ concentrations were described in previous studies (Espino et al., 2010; Akpınar et al., 2016), assuming a Kd of 155 nM. The [Ca^2+^]_i_ concentrations in TRPM2 and TRPV1 experiments were recorded by using the integral of the rise in [Ca^2+^]_i_ for 125 s after addition of cumene hyroperoxide (CPx and 0.1 mM) or capsaicin (0.01 mM) (Akpınar et al., 2016; Demirdaş et al., 2017), respectively. The [Ca^2+^]_i_ concentration is expressed as nanomolar (nM) taking a sample every second as previously described (Espino et al., 2010).

### Electrophysiology

We used whole-cell mode of patch-clamp techniques (EPC10 patch-clamp set, HEKA, Lamprecht, Germany) was used in the DRGN of current studies (Akpınar et al., 2016; Özdemir et al., 2016). Resistances of whole cell recording electrodes were adjusted to about 3–6 MΩ by a puller (PC-10 Narishige International Limited, London, UK). We used standard extracellular bath and pipette solutions as described in previous studies (Nazıroğlu and Lückhoff, 2008; Akpınar et al., 2016). The intracellular Ca^2+^ concentration was held as 1 μM instead of physiological concentration (0.1 μM) in TRPM2 experiments because the channels are activated by presence of high intracellular Ca^2+^ concentration (McHugh et al., 2003). Holding potential of the patch-clamp analyses in the DRGNs was −60 mV. Voltage clamp technique was used in the analyses and current-voltage (I-V) relationships were obtained from voltage ramps from −90 to +60 mV applied over 200 ms. All experiments were performed at room temperature (22 ± 1°C).

In the experiments, TRPM2 channels are gated by ADPR (1 mM in patch pipette) although they were inhibited by ACA (0.025 mM). In a recent study, we observed activation of TRPV1 channels by medium level (0.01 mM) CAPS instead of low (0.001 mM) CAPS (Nazıroğlu, 2017). Therefore, TRPV1 channels were activated by adding extracellular (in patch chamber) CAPS (0.010 mM), and the channels were inhibited by administration of capsazepine (CapZ and 0.1 mM) into patch chamber through extracellular buffer. For the analysis, the maximal current amplitudes (pA) in a DRGN were divided by the cell capacitance (pF), a measure of the cell surface. The results in the patch clamp experiments are the current density (pA/pF).

### Intracellular ROS production measurement

Dihydrorhodamine (DHR) 123 is an uncharged and nonfluorescent intracellular ROS production indicator. It can easily pass across cell membranes where it is oxidized to cationic rhodamine 123 which localizes in the mitochondria and exhibits green fluorescence. The sciatic nerve and DRGNs were incubated with 20 μm DHR 123 at 37°C for 25 min (Bejarano et al., 2009). The fluorescence intensities of the rhodamine 123 were assayed (excitation; 488 nm and emission; 543 nm) by using an automatic microplate reader (Infinite pro200; Tecan Inc, Groedig, Austria). The results were expressed as fold-increase over the pretreatment level.

### Mitochondrial membrane potential (JC-1) analyses

The mitochondrial membrane potential (5,5′,6,6′-tetrachloro-1,1′,3,3′-tetraethylbenzimidazolocarbocyanine iodide, JC-1) was determined by JC-1 dye as described in previous studies (Bejarano et al., 2009; Espino et al., 2010). The JC-1- loaded sciatic and DRGNs neurons at 37°C for 45 min were excited at 488 nm and emission was detected at 590 nm (JC-1 aggregates) and 525 nm (JC-1 monomers). Values were calculated from emission ratios (590/525) and they are presented as fold-increase.

### Cell viability assay

To determine the cell viability after SNI induction and HP treatment, we used to cell viability analyses as 3-(4,5-Dimethylthiazol-2-yl)-2,5-diphenyltetrazolium bromide (MTT) in the neurons as described elsewhere (Demirdaş et al., 2017). After incubation for 60 min with medium containing MTT solution (5 mg/ml), removed the neurons and dissolved the resulting MTT formazan in DMSO. Absorbance values were recorded in a spectrophotometer at 490 nm (UV-1800, Shimadzu, Kyoto, Japan). The data are presented as the fold increase over the pretreatment level (experimental/control).

### Assay for apoptosis, caspase 3, and 9 activities

The apoptosis levels were determined by using the spectrophotometer and a commercial kit of Biocolor Ltd. (Northern Ireland) as described in a previous study (Demirdaş et al., 2017). The method is based on loss of asymmetry in membranes of apoptotic neurons.

The determinations of caspase 3 and caspase 9 activities in the sciatic nerve and DRGN neurons were performed in the microplate reader (Infinite pro200) by using caspase 3 (N-acetyl-Asp-Glu-Val-Asp-7-amino-4-methylcoumarin) and caspase 9 (His-Asp-7-amino-4-methylcoumarin) substrates. Details of the assays were indicated in recent studies (Akpınar et al., 2016; Özdemir et al., 2016). The substrate cleavage was measured at 360 nm (excitation) and 460 nm (emission). Values were calculated as fluorescence units/mg protein. The data are expressed as fold-increase.

### Western blot analyses

Standard procedures are used in the Western Blot analyses of sciatic nerve, DRGN, muscle, and skin (Akpınar et al., 2016; Özdemir et al., 2016). In the analyses, caspase 9 (p35/p10 Polyclonal Antibody), caspase 3 (p17-specific Polyclonal Antibody), beta actin (polyclonal antibody) and Poly-ADPR polymerase 1 (PARP-1) (polyclonal antibody) were purchased from (Proteintech, USA) although secondary antibodies (Rabbit IgG, HRP-linked whole anti-Aβ, from donkey) were purchased from GE Healthcare (Amersham, UK). Relative levels of immunoreactivity in ECL Western HRP Substrate (Millipore Luminate Forte, USA) were quantified using Syngene G:Box Gel Imagination System (UK). Rabbit anti-β-actin (1:2000) was used as an internal control for the concentration of proteins loaded. The data are expressed as relative density over the control level.

### Statistical analyses

All data were represented as means ± standard deviation (SD). The data were analyzed by using 17.0 version of SPSS statistical program (Chicago, Illinois, USA). *P* ≤ 0.05 was considered to indicate a statistically significant difference. Presence of significance in the five groups was once detected by LSD-test. Then *p*-value levels of significances in the data were analyzed by using Mann-Whitney *U*-test.

## Results

### Effects of HP on TRPM2 channel activation-induced [Ca^2+^]_i_ concentration in sciatic nerve and DRGNs of SNI-induced rats

**Figure 2:**
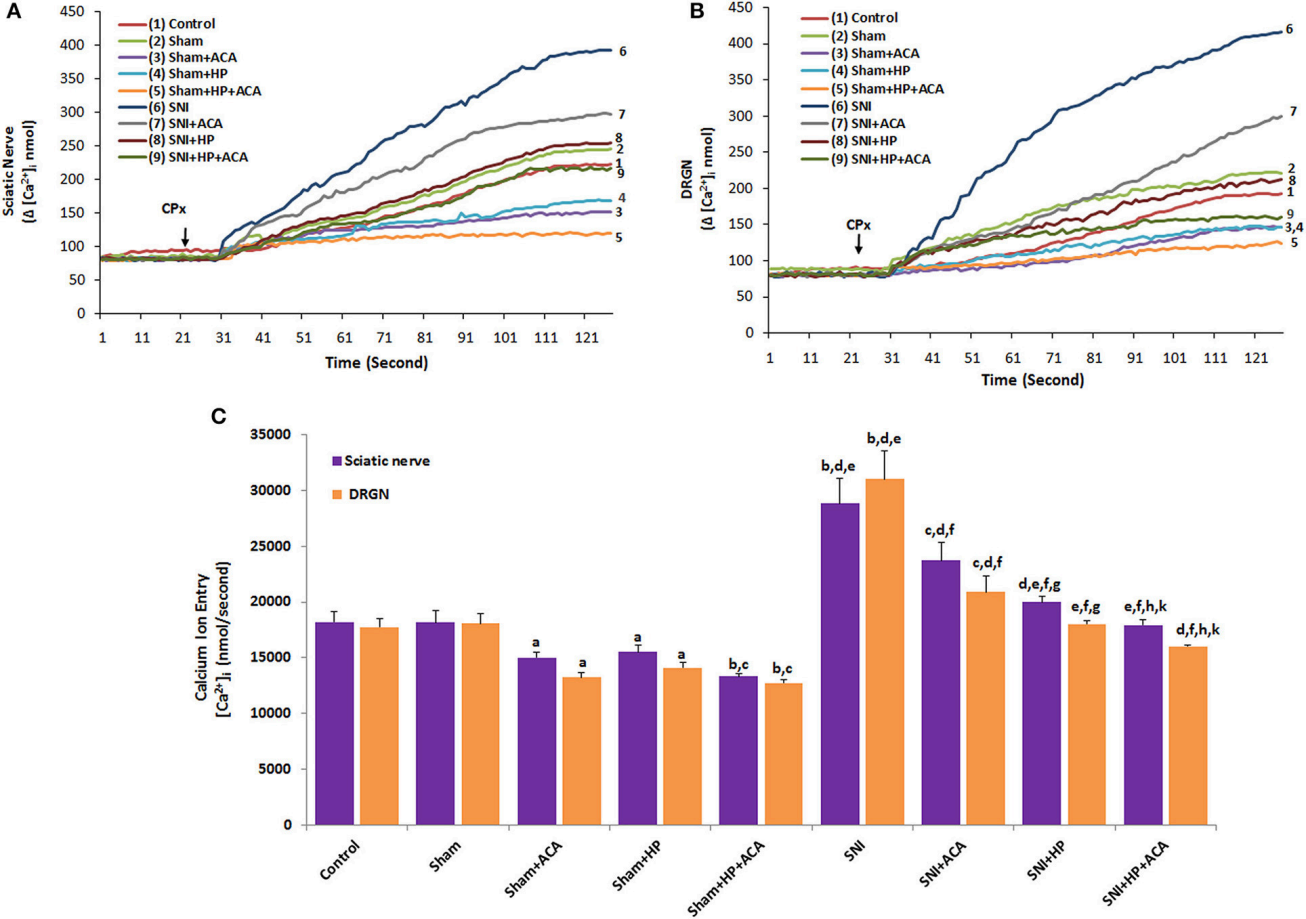
**Effect of ***Hypericum perforatum*** (HP) treatment on [Ca^**2+**^]_**i**_ concentration and TRPM2 in sciatic nerve (A)** and dorsal root ganglion neurons (DRGNs) **(B)** of control, sham and SNI-induced rats. (*n* = 8 and mean ± SD). The animals received oral HP for 4 weeks. Then, these dissected neurons of control, sham and SNI were further *in vitro* treated with CPx (0.1 mM) and ACA (0.025 mM) before loading Fura-2 for 125 s. ^a^*p* ≤ 0.05 and ^b^*p* ≤ 0.001 vs. control and sham groups. ^c^*p* ≤ 0.05 and ^d^*p* ≤ 0.001 vs. sham+ACA and sham+HP groups. ^e^*p* ≤ 0.001 vs. sham+HP+ACA group. ^f^*p* ≤ 0.001 vs. SNI group. ^g^*p* ≤ 0.05 and ^h^*p* ≤ 0.001 vs. SNI+ACA group. ^k^*p* ≤ 0.05 vs. SNI+HP group **(C)**.

TRPM2 channel was discovered as first candidate of oxidative stress dependent TRP channels because it has oxidative sensitive ADPR pyrophosphatase enzyme in C domain (Perraud et al., 2001). HP is containing antioxidant flavonoids in its content (Kusari et al., 2009; Stojanović et al., 2013). In etiology of SNI, oxidative stress has main role and HP may modulate the oxidative stress dependent-activated TRPM2 in sciatic nerve and DRGN of SNI-induced rats. For clarifying the modulator role of HP in the neurons, the neurons of HP supplemented rats were further *in vitro* stimulated by CPx (0.1 mM) (Figures 2A,B). Addition of CPx caused a significant rise in [Ca^2+^]_i_ concentration of sciatic nerve and DRGNs of SNI group which is attributed to activation of Ca^2+^-permeable TRPM2 channels. This rise in intracellular [Ca^2+^]_i_ concentration was markedly (*p* ≤ 0.001) higher in the SNI group than in the control and sham groups (Figure 2C). We observed low level of **[**Ca^2+^]_i_ concentration of the neurons in HP and ACA treated group. The [Ca^2+^]_i_ concentration in the neurons was significantly lower in the sham+HP (*p* ≤ 0.05), sham+ACA (*p* ≤ 0.05), and sham+HP+ACA (*p* ≤ 0.001) groups than in control and sham groups. The [Ca^2+^]_i_ concentration in the neurons was low in SNI+HP and SNI+ACA and SNI+HP+ACA groups as compared to as compared to SNI only (*p* ≤ 0.001). It seems that HP modulated the SNI-overload [Ca^2+^]_i_ concentration through regulation of TRPM2 in the neurons.

### Effects of HP on TRPV1 channel activation-induced [Ca^2+^]_i_ concentration in sciatic nerve and DRGNs of SNI-induced rats

**Figure 3:**
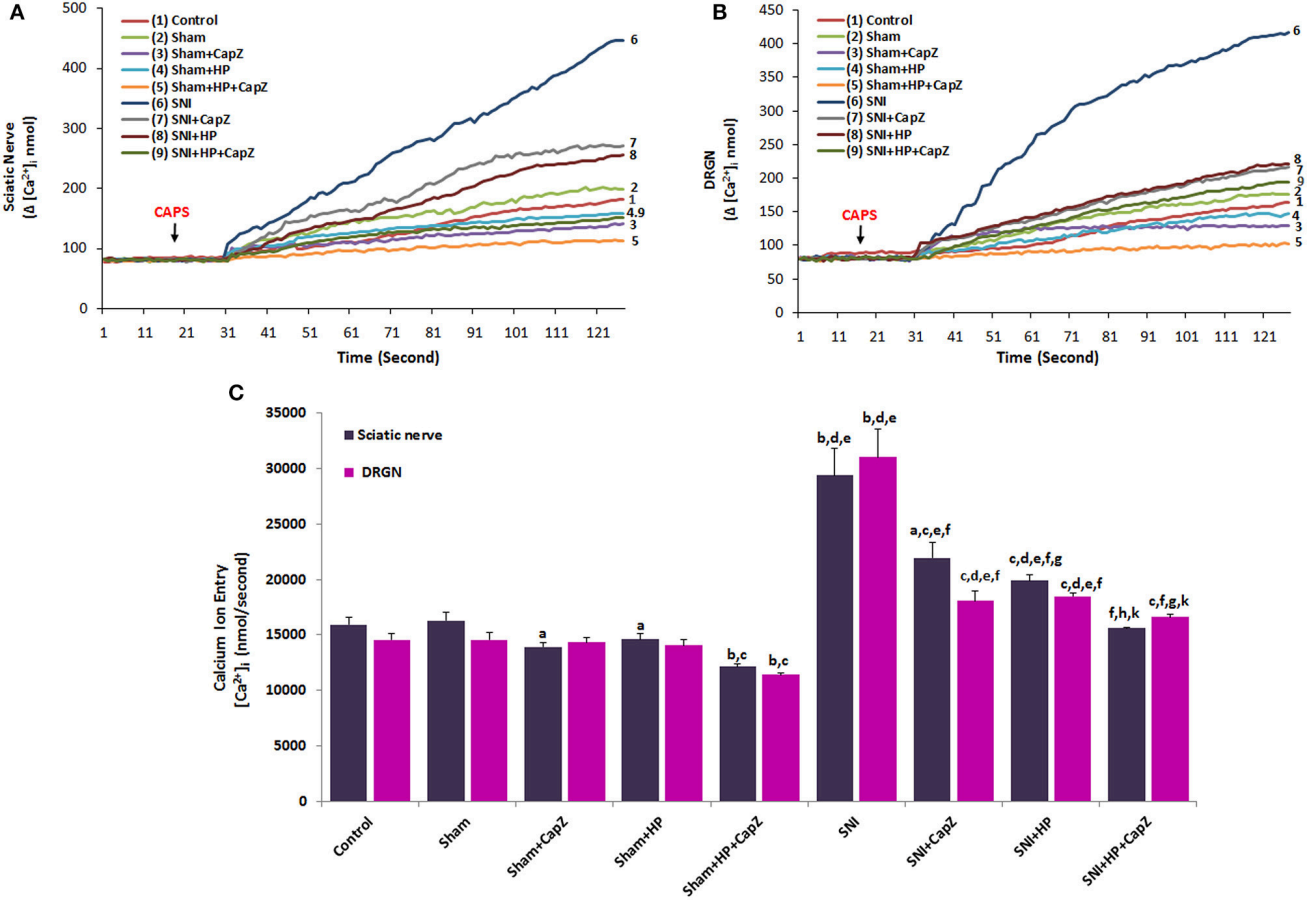
**Effect of ***Hypericum perforatum*** (HP) treatment on [Ca^**2+**^]_**i**_ concentration and TRPV1 in sciatic nerve (A)** and dorsal root ganglion neuron (DRGN) **(B)** of control, sham and SNI-induced rats. (*n* = 8 and mean ± SD). The animals received oral HP for 4 weeks. Then, these dissected neurons of control, sham and SNI were further *in vitro* treated with CAPS (0.01 mM) and CapZ (0.1 mM) before loading Fura-2 for 125 s. ^a^*p* ≤ 0.05 and ^b^*p* ≤ 0.001 vs. control and sham groups. ^c^*p* ≤ 0.05 and ^d^*p* ≤ 0.001 vs. sham+CapZ and sham+HP groups. ^e^*p* ≤ 0.001 vs. sham+HP+CapZ group. ^f^*p* ≤ 0.001 vs. SNI group. ^g^*p* ≤ 0.05 and ^h^*p* ≤ 0.001 vs. SNI+CapZ group. ^k^*p* ≤ 0.05 vs. SNI+HP group **(C)**.

Stimulation of CAPS caused a significant rise in [Ca^2+^]_i_ concentration of sciatic nerve and DRGNs of SNI group which is attributed to activation of Ca^2+^-permeable TRPV1 channels (Figures 3A,B). Figure 3C showed that, comparing with the control and sham groups, despite of the fact that the concentration of [Ca^2+^]_i_ was higher in SNI group, the TRPV1 inhibitors (HP and CapZ) could efficiently decrease the concentration of [Ca^2+^]_i_ which was induced by SNI induction (*p* ≤ 0.001). We observed low level of **[**Ca^2+^]_i_ concentration of the neurons in HP and CapZ treated group. The [Ca^2+^]_i_ concentration in the neurons was significantly lower in the sham+HP (*p* ≤ 0.05), sham+CapZ (*p* ≤ 0.05) and sham+HP+CapZ (*p* ≤ 0.001) groups than in control and sham groups. The [Ca^2+^]_i_ concentration in the neurons was low in SNI+HP and SNI+CapZ and SNI+HP+CapZ groups as compared to as compared to SNI only (*p* ≤ 0.001). It seems that HP modulated the SNI-overload [Ca^2+^]_i_ concentration through regulation of TRPV1 in the neurons.

### Effects of HP on ADPR-induced TRPM2 currents in DRGN of SCI-induced rats

**Figure 4:**
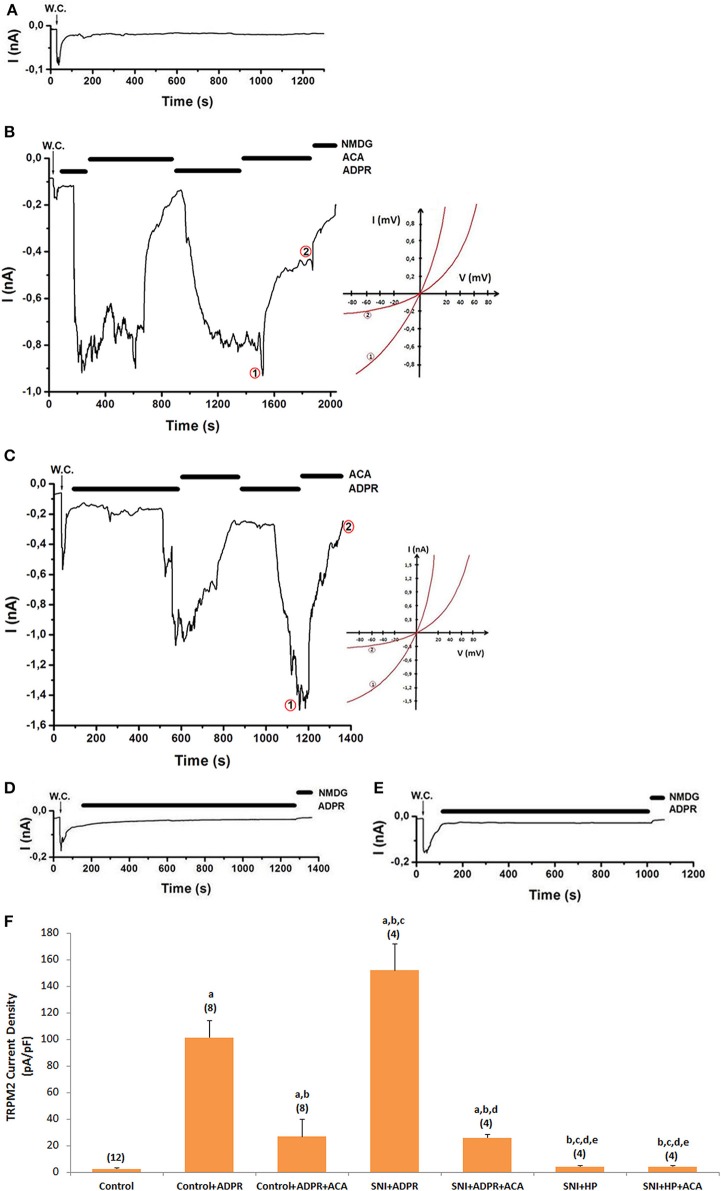
**Effects of HP on TRPM2 channel activation in dorsal root ganglion neuron (DRGN) of control and SNI-induced rat**. The TRPM2 currents in DRGN were stimulated by intracellular ADPR (1 mM in patch pipette) but they were inhibited by extracellular ACA (0.025 mM) in the patch-chamber. W.C.: Whole cell. Control (without SCI induction and stimulation): Original recordings from control neuron **(A)**. **(B)**. Control+ADPR group (without SCI induction). **(C)**. SNI group (with SCI induction). **(D)**. SCI+HP group: The rats received HP after SCI induction. **(E)**. HP group: The rats received HP without SCI induction. **(F)**. TRPM2 channel current densities in the DRGN. The numbers in parentheses indicated n numbers of groups were indicated by numbers in parentheses. (^a^*p* ≤ 0.001 vs. control. ^b^*p* ≤ 0.001 vs. control+ADPR group. ^c^*p* ≤ 0.001 vs. control+ADPR+ACA group. ^d^*p* ≤ 0.001 vs. SNI+ADPR group. ^e^*p* ≤ 0.001 vs. SNI+ADPR+ACA group).

The effects of antioxidant HP for TRPM2 channels activated by ADPR are indicated in Figure 4. ADPR (1 mM) induced a current in murine DRGNs (Figures 4B,C). There was no current in absence of ADPR (Figure 4A). Current densities of the DRGNs were markedly (*p* ≤ 0.001) higher in the SNI+CAPS group (Figure 4F) than in the control (*p* ≤ 0.001), control+CAPS (*p* ≤ 0.05) and control+CAPS+CapZ (*p* ≤ 0.001) groups. There was no activation of TRPM2 channel in HP (Figure 4D) and SNI+HP (Figure 4E) groups and the current densities in the DRGNs were significantly (*p* ≤ 0.001) lower in HP and SCI+HP groups as compared to the SCI group (Figure 3). These results indicate that up-regulation of TRPM2 channel activity through HP treatment may be critical for SNI-mediated overload Ca^2+^ entry and intracellular ROS production in the DRGNs.

### Effects of HP on CAPS-induced TRPV1 currents in DRGNs of control and SNI-induced rats

**Figure 5:**
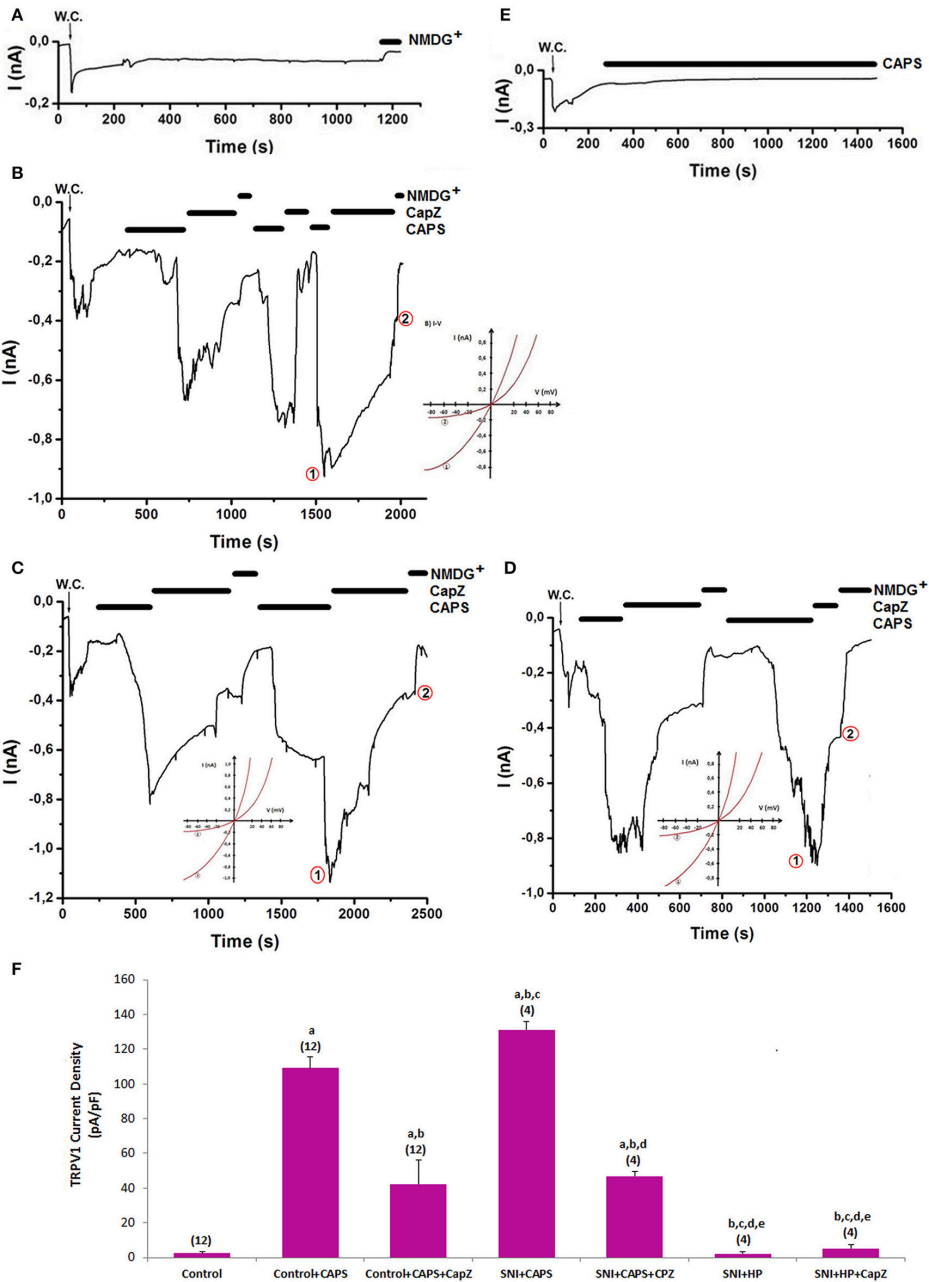
**Effects of HP on TRPV1 channel activation in dorsal root ganglion neuron (DRGN) of control and SNI-induced rat**. The TRPV1 currents in DRGN were stimulated by extracelular capsaicin (CAPS and 0.01 mM in patch chamber) but they were inhibited by extracellular CaPZ (0.1 mM) in the patch-chamber. W.C.: Whole cell. Control (without SCI induction and stimulation): Original recordings from control neuron **(A)**. **(B)**. Control+CAPS group (without SCI induction). **(C)**. SNI group (with SCI induction). **(D)**. SCI+HP group: The rats received HP after SCI induction. **(E)**. HP group: The rats received HP without SCI induction. **(F)**. TRPV1 channel current densities in the DRGN. The numbers in parentheses indicated n numbers of groups were indicated by numbers in parentheses. (^a^*p* ≤ 0.001 vs. control. ^b^*p* ≤ 0.001 vs. control+CAPS group. ^c^*p* ≤ 0.001 vs. control+CAPS+CapZ group. ^d^*p* ≤ 0.001 vs. SNI+ADPR group. ^e^*p* ≤ 0.001 vs. SNI+ADPR+ACA group).

The murine DRGNs were activated by capsaicin (Figures 5B–D). The CAPS-induced currents were reversibly and partially blocked by CapZ and NMDG^+^ replacement instead of Na^+^. There was no current in the absence of CAPS (Figure 5A). The current densities of DRGNs were significantly higher in the SNI+CAPS group than in control (*p* ≤ 0.001) and control+CAPS (*p* ≤ 0.05) groups although the densities were significantly (*p* ≤ 0.001) lower in control+CAPS+CapZ and SNI+CAPS+CapZ groups as compared to in the SNI groups (Figures 5E,F). The densities were decreased in the neuron by HP treatment and they were low in SNI+HP and SNI+HP+CapZ groups (*p* ≤ 0.001). These results indicate that CAPS and ROS overload the Ca^2+^ entry through TRPV1 channel activation. However, the SNI-induced TRPV1 currents through modulation of oxidative stress were decreased by the antioxidant HP treatments.

### Effect of HP on the apoptosis and cell viability (MTT) values in the SNI-induced sciatic nerve and DRGNs

**Figure 6:**
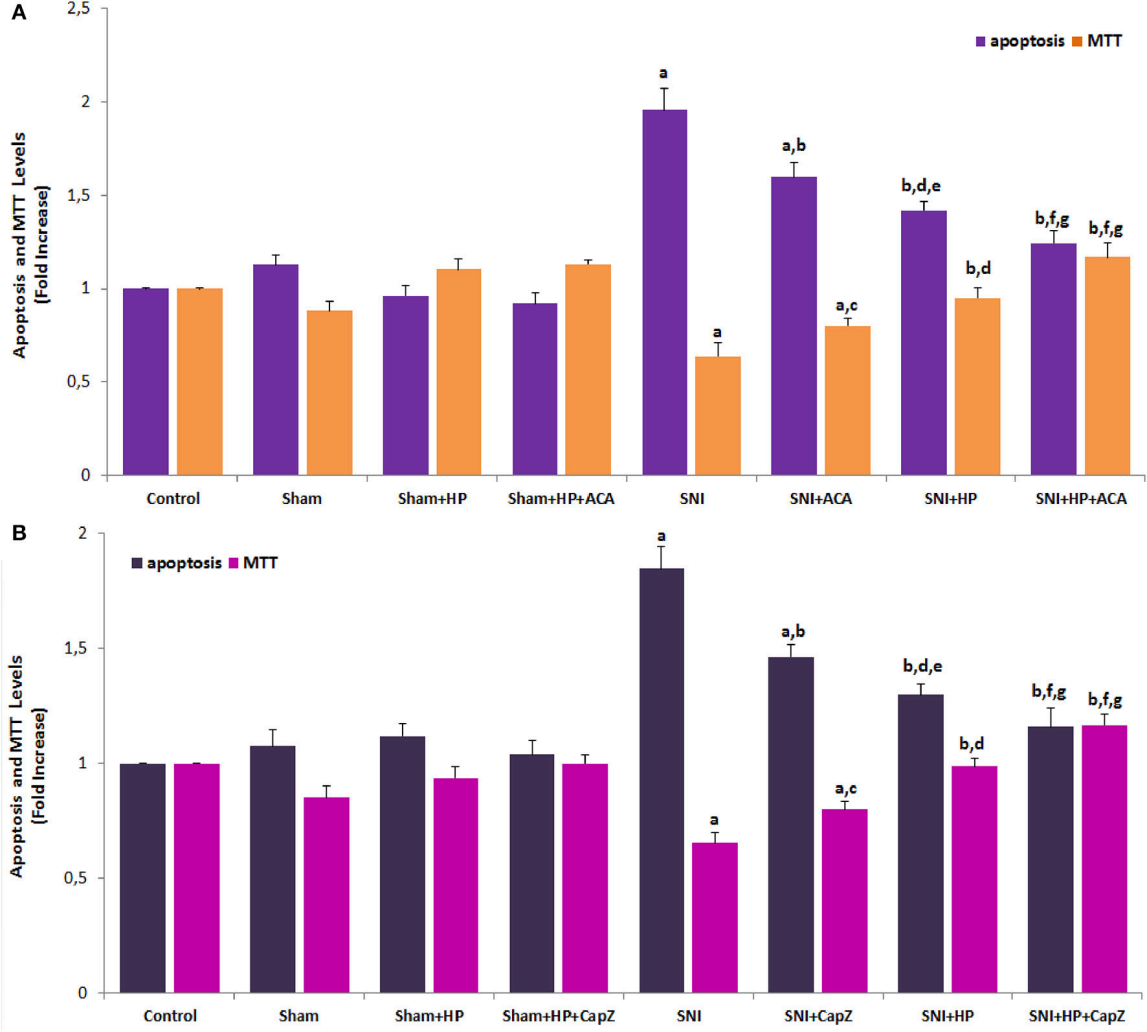
**Effects of ***Hypericum perforatum*** (HP) on the apoptosis and cell viability (MTT) levels through TRPM2 (A)** and TRPV1 **(B)** in sciatic nerve of SNI-induced rats (mean ± SD and *n* = 3). Apoptosis level was measured by using a commercial kit. Values expressed as fold increase (experimental/control). These neurons were dissected from control, SNI and treated animals. The animals were received HP via gastric gavage. The neurons in TRPM2 and TRPV1 experiments were stimulated with cumene hydroperoxide (CPx and 0.1 mM) capsaicin (CAPS and 0.01 mM) although they were inhibited by ACA (0.025 mM) and CapZ (0.1 mM), respectively. (^a^*p* ≤ 0.001 and ^e^*p* ≤ 0.05 vs. control, sham, sham+HP, sham+HP+ACA and sham+HP+CapZ groups. ^b^*p* ≤ 0.001 and ^c^*p* ≤ 0.05 vs. SNI group. ^d^*p* ≤ 0.05 and ^f^*p* ≤ 0.001 vs. SNI+ACA and SNI+CapZ groups. ^g^*p* ≤ 0.05 vs. SNI+HP group).

Involvements of TRPM2 and TRPV1 on the apoptosis and MTT in the sciatic nerve and DRGN are shown in Figures 6A,B, respectively. Apoptosis levels were markedly (*p* ≤ 0.001) measured high in the SNI group, although MTT-values were significantly (*p* ≤ 0.001) lower in the SNI group. However, the apoptosis levels were markedly decreased in ACA (*p* ≤ 0.05), CapZ (*p* ≤ 0.05), and HP (*p* ≤ 0.001) treated groups although MTT-values were (*p* ≤ 0.05 and *p* ≤ 0.001) increased by the treatments.

### Effect of HP on the caspase activities, intracellular ROS production and JC-1 level in the sciatic nerve of control, SNI and HP groups

**Figure 7:**
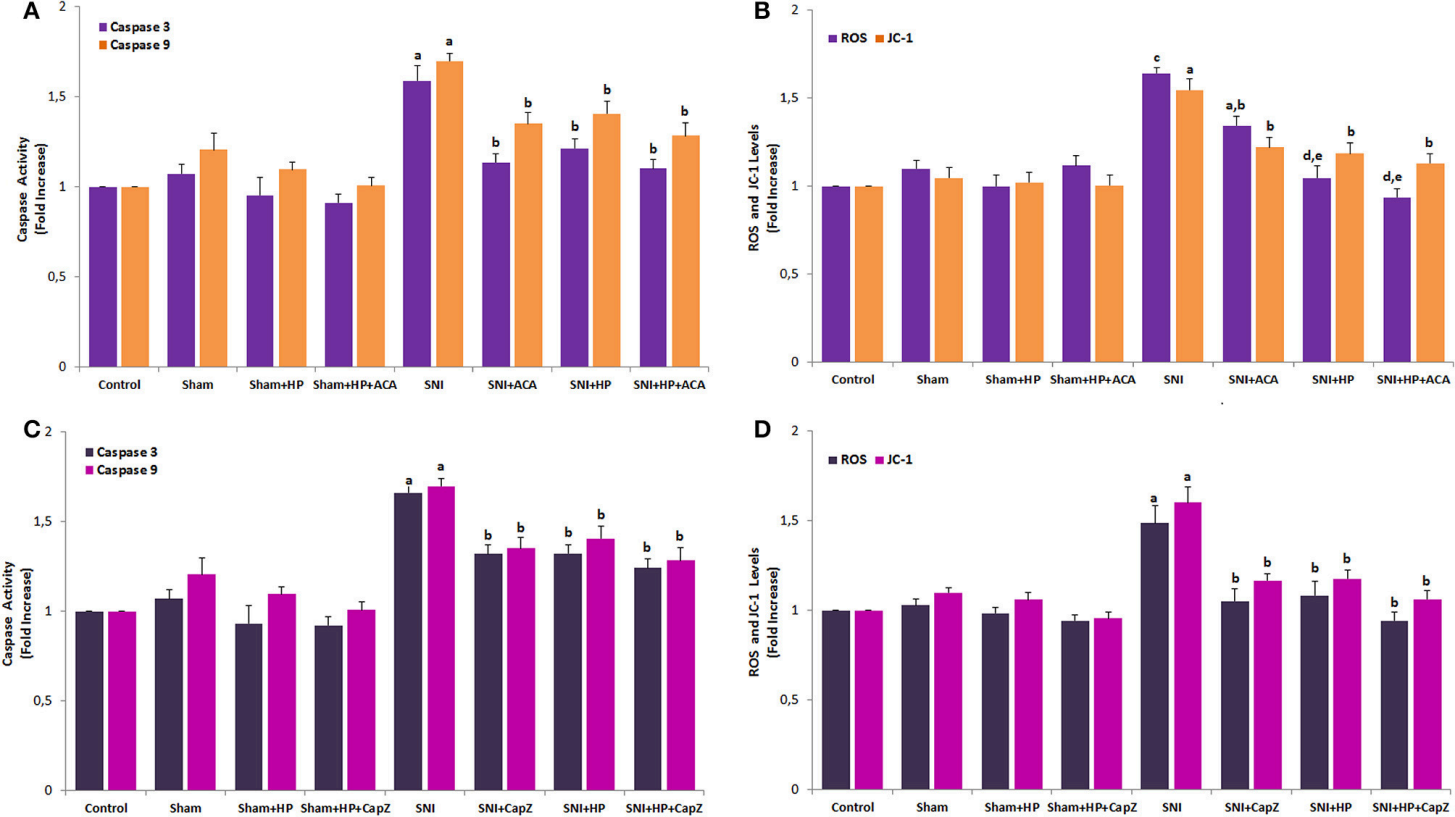
**Effects of ***Hypericum perforatum*** (HP) on the intracellular ROS production and cell mitochondrial membrane depolarization (JC-1) levels through TRPM2 (A,C)** and TRPV1 **(B,D)** in sciatic nerve of SNI-induced rats (mean ± SD and *n* = 3). Values expressed as fold increase (experimental/control). Sciatic neurons were dissected from control, SNI and HP treated animals. The neurons in TRPM2 and TRPV1 experiments were stimulated with cumene hydroperoxide (CPx and 0.1 mM) capsaicin (CAPS and 0.01 mM) although they were inhibited by ACA (0.025 mM) and CapZ (0.1 mM), respectively. (^a^*p* ≤ 0.05 and ^c^*p* ≤ 0.001 vs. control, sham, sham+HP, sham+HP+ACA and sham+HP+CapZ groups. ^b^*p* ≤ 0.05 and ^d^*p* ≤ 0.001 vs. SNI group. ^e^*p* ≤ 0.05 vs. SNI+ACA group).

Caspase activity analyses were performed caspase 3 and 9 substrate in the plate reader. The caspase 3 and 9 activities were markedly (*p* ≤ 0.05) increased in sciatic nerve and DRGNs (data are not shown) of SNI groups through TRPM2 (Figure 7A) and TRPV1 (Figure 7C) activations. However, the caspase activities were markedly (*p* ≤ 0.05) decreased in the neurons through inhibition of TRPM2 and TRPV1 channels by HP with/without ACA and CapZ treatments.

Induction of SCI in rats induced a mitochondrial membrane depolarization as detected by the increase in the mitochondrial-specific voltage-sensitive dye JC-1 fluorescence ratio. The JC-1-value in the sciatic nerve (Figures 7B,D) and DRGNs (unpublished data) was significantly (*p* ≤ 0.05) higher in the SNI group than in the control and HP groups although its value was significantly (*p* ≤ 0.01) lower in the SNI+ACA, SNI+CapZ, SNI+HP, SNI+HP+ACA, and SNI+HP+CapZ groups than in the SNI group only.

Previous studies demonstrated that DRGNs produced intracellular ROS under nerve injuries through TRPM2 and TRPV1 channel activations (Ding et al., 2016; Özdemir et al., 2016). To determine whether HP, as an antioxidant plant extract, can cause redundant ROS accumulation in cytosol of sciatic nerve and DRGNs, we investigated intracellular ROS levels through TRPM2 and TRPV1 channel activations in SNI-induced and HP-treated sciatic nerve and DRGNs. The SNI-induced increase of intracellular ROS level in SNI group was also decreased in the SNI groups by HP, ACA and CapZ treatment (*p* ≤ 0.05). The results implied that HP treatments might decrease the levels of SNI-induced mitochondrial ROS in the sciatic nerve and DRGNs by inhibiting TRPM2 and TRPV1. The JC-1 and ROS levels were further decreased in SNI+ACA (*p* ≤ 0.05 and (*p* ≤ 0.001) and SNI+HP+ACA (*p* ≤ 0.05 and (*p* ≤ 0.001) groups as compared to SNI and SNI+HP groups. Therefore, involvement of TRPM2 channel inhibition on the JC-1 and ROS in the sciatic nerve was more significant than in inhibition of TRPV1 channels due to antioxidant properties of HP.

### Effect of HP on PARP-1, caspase 3, and 9 expression levels in sciatic nerve, DRGN, skin, and muscle of the SCI-induced rats

**Figure 8:**
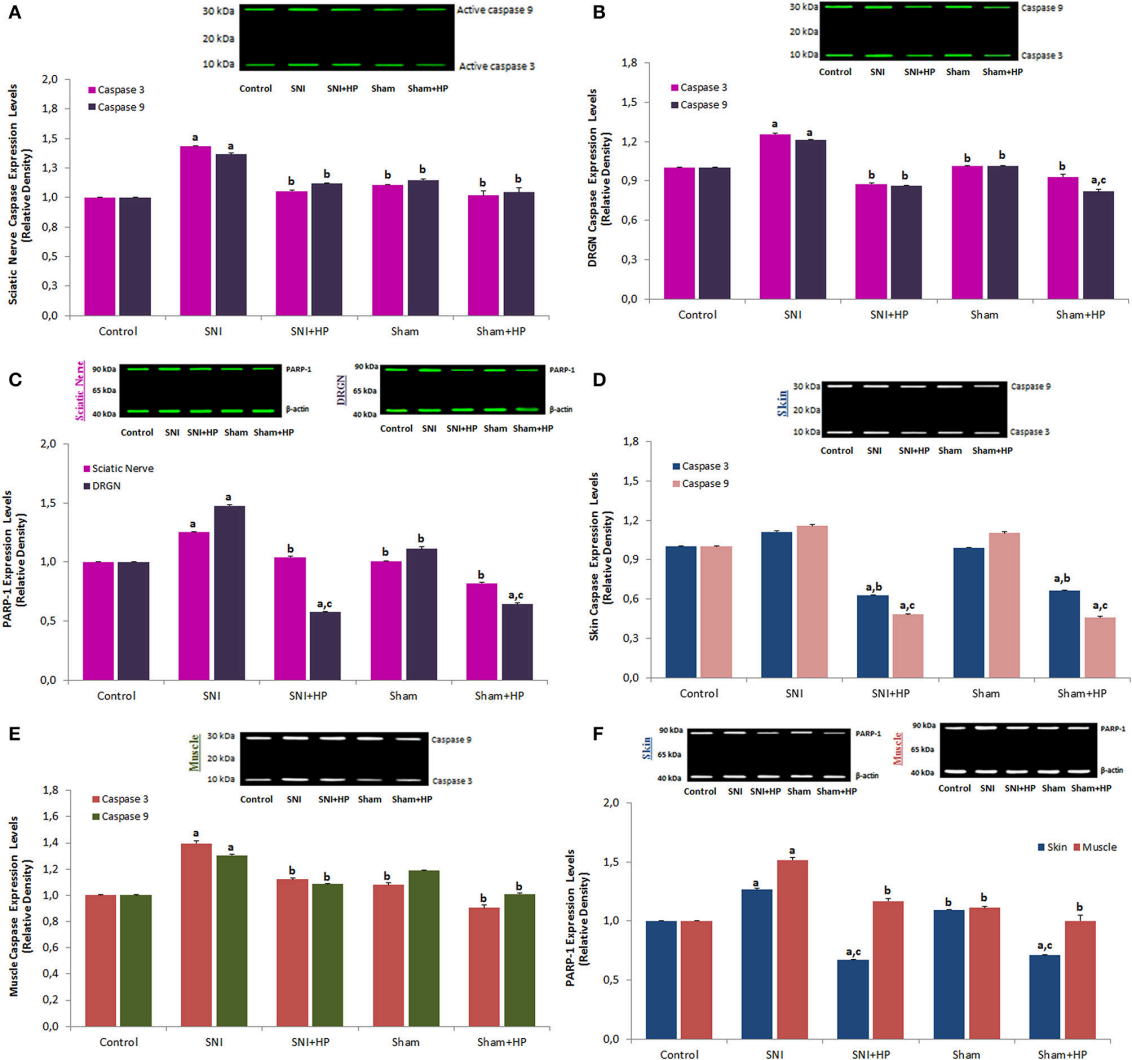
**Effects of ***Hypericum perforatum*** (HP) on the PARP-1, caspase 3 and 9 expression levels in sciatic nerve (A,C)**, DRGN **(B,C)**, skin **(D,F)** and muscle (Musculus piriformis) **(E,F)** and skin **(F)** of rats with SNI (mean ± SD and *n* = 3). Anti-β-actin was used as an internal control for the concentration of PARP1, caspase 3 and 9. Values expressed as fold increase (experimental/control). Sciatic nerve DRGN neurons were dissected from control, SNI and HP treated animals. (^a^*p* ≤ 0.05 vs. control, sham, sham+HP groups. ^b^*p* ≤ 0.05 and ^c^*p* ≤ 0.001 vs. SNI group).

Caspase 3 is synthesized as an inactive pro-enzyme that is processed in cells undergoing apoptosis by self-proteolysis and/ cleavage by other caspase activation, including caspase 9. The caspase 9 is activated by the active caspase 3 (Carrasco et al., 2015). Caspase 9 induces death signals by triggering other types of caspase activation. Active caspase 3 and 9 expression levels act main role the progress of apoptosis in neuronal injury (Özdemir et al., 2016). In the current study, Caspase 3 and 9 expression levels in the sciatic nerve (Figure 8A), DRGN (Figure 8B), skin (Figure 8D), and muscle (Figure 8E) were markedly (*p* ≤ 0.05) higher in SNI group than in control. However, the caspase expression levels in the four samples were decreased by the HP treatments and their expression levels in the sciatic nerve (*p* ≤ 0.05), DRGN (*p* ≤ 0.05), skin (*p* ≤ 0.001), and muscle (*p* ≤ 0.05) were markedly lower in SNI+HP and sham+HP groups than in SNI group only.

PARP-1 acts main role in DNA repair (Nazıroğlu, 2007) and its expression level is increased in neurodegenerative diseases such as SCI and SNI but it expression level was decreased in SNI and DRGN by antioxidants (Wu et al., 2009; Yin et al., 2015). PARP-1 is also acted a source for many apoptotic proteases, including caspase 3 (Citron et al., 2000). In the current study, we analyzed PARP-1 expression levels in the sciatic nerve (Figure 8C), DRGN (Figure 8C), skin and muscle (Figure 8F). PARP-1 expression levels in the sciatic nerve, DRGN, skin and muscle were markedly (*p* ≤ 0.05) higher in SNI group as compared to control. However, the PARP-1 levels in the sciatic nerve (*p* ≤ 0.05), DRGN (*p* ≤ 0.001), skin (*p* ≤ 0.001), and muscle (*p* ≤ 0.05) were markedly lower in SNI+HP and sham+HP groups as compared to SNI group only.

## Discussion

The current results implied that HP treatments might decrease the levels of SNI-induced [Ca^2+^]_i_ accumulation, mitochondrial ROS, apoptosis levels, and PARP-1, caspase 3, 9 activities and expressions in the sciatic nerve and DRGNs by inhibiting TRPM2 and TRPV1. To our knowledge, this is the first evidence for a function of SNI pathophysiological process implicating the sciatic nerve and DRGN and, in particular, peripheral pain, and neurodegenerative diseases.

Recent reports indicate that functional TRPM2 and TRPV1 are expressed in the sciatic nerve and DRGNs (Isami et al., 2013; Pecze et al., 2013; Akpınar et al., 2016), the present literature findings suggest that TRPM2 and TRPV1 act role in acute mechanical nociceptive pain Ca^2+^ signaling. Considerable evidence indicated that TRPM2 and TRPV1 are activated and potentiated by excessive intracellular ROS production (Susankova et al., 2006; Ding et al., 2016). Activation of TRPM2 and TRPV1 enhanced [Ca^2+^]_i_ accumulation due to their permeability to Ca^2+^ (Pecze et al., 2013, 2016; Nazıroğlu et al., 2014a) which were involved in several physiological and pathological processes such as neuronal viability, apoptosis, and neuronal recovering signaling. The SCI-induced oxidative stress status evokes TRPM2 and TRPV1 channels to activation and triggers higher amounts of Ca^2+^ entry to the cell cytosol (Özdemir et al., 2016). HP is strong antioxidant because it contains several flavonoid antioxidants (Kusari et al., 2009; Stojanović et al., 2013). As source of these antioxidants, HP acts important role in etiology of neurodegenerative diseases such as SNI and SNI (Kusari et al., 2009; Stojanović et al., 2013). Recent studies have observed perturbations of Ca^2+^ homeostasis through TRPM2 and TRPV1 activations caused by excessive levels of mitochondrial oxidative stress in the neurons from experimental animals with nerve injury (Nazıroğlu et al., 2014a; Xiang et al., 2016). Induction of SNI elevates oxidative stress levels in neurons (Rogoz et al., 2015) and consequence of excessive Ca^2+^ influx, apoptosis exists by activation of cation channels (Özdemir et al., 2016). In the current study, we observed SNI-induced [Ca^2+^]_i_ accumulation and increased current densities through TRPM2 (ADPR and CPx) and TRPV1 (CAPS) channel activators caused by excessive levels of mitochondrial oxidative stress, although their levels were decreased by antioxidant property of HP.

We found also that the level of Ca^2+^ influx through the inhibition of TRPM2 and TRPV1 channels decreased by the HP treatment. It is well-known that intracellular Ca^2+^ signaling with/without oxidative stress acts an important role in pathophysiological functions of pain. Increases in Ca^2+^ concentration may conduce to the membrane mitochondrial depolarization (Bejarano et al., 2009; Espino et al., 2010), activation of ADPR pyrophosphatase that will enhance the TRPM2 channel potency, and activation of a variety of intracellular enzymes such as PARP-1 and caspase (Perraud et al., 2001; Hara et al., 2002; Nazıroğlu and Lückhoff, 2008). Previous studies have shown that the intracellular Ca^2+^ influx into sciatic nerve and DRGNs neurons through increased activity sensitization of TRPM2 and TRPV1 channels acted a main role in mechanical hypersensitivity and pain associated with nerve injury (Haraguchi et al., 2012; Nazıroğlu, 2012, 2015; Rogoz et al., 2015) although the hypersensitivity and pain are decreased by inhibition of calcium channels through HP treatment (Uchida et al., 2008; Nazıroğlu et al., 2014a; Özdemir et al., 2016). Hence, we provided the novel finding that HP treatment potently decreased SNI-induced overload intracellular Ca^2+^ entry by modulation of TRPM2 and TRPV1 channel activations.

The impairment of neuronal membrane permeability causes overload Ca^2+^ influx into cytosol and it leads to excessive production of ROS in the neurons (Kumar et al., 2014; Demirdaş et al., 2017). Increased [Ca^2+^]_i_ concentration through activation of TRPM2 and TRPV1 causes disruption of the Ca^2+^ contents of intermembrane space through mitochondrial permeability transition activation in the mitochondria (Pecze et al., 2013). The dysfunction of mitochondria triggers generation of endogenous ROS. Caspases, a group of enzymes are activated by overload [Ca^2+^]_i_ concentration and excessive ROS products that found cleavage (inactive) caspases before the neurons undergo apoptosis (Citron et al., 2000). However, taken together the excessive ROS production and Ca^2+^ impairment of the neurological cells have revealed that a key role in the pathogenesis of neurodegenerative diseases such as SNI and SCI (Gupta et al., 2014; Özdemir et al., 2016). Antioxidants through inhibition of TRPM2 and TRPV1 regulate the mitochondrial and apoptotic imbalance and help to normal neuronal functions (Nazıroğlu, 2012). In the current study, the apoptosis, caspase 3, caspase 9, PARP-1, JC-1, and intracellular ROS-values were increased in the sciatic nerve and DRGNs by SNI induction although their values were decreased in the neurons by HP, TRPM2 (ACA), and TRPV1 (CapZ) blockers. Similarly, apoptosis, ROS, JC-1, caspase 3 and 9 values through inhibition of TRPM2 in human phagocytic cells were decreased by HP incubations (Nazıroğlu et al., 2014b,c). The modulator role of HP on TRPM2 channels and oxidative stress in DRGN of rats was indicated by an experimental rat study (Nazıroğlu et al., 2014a). More recently, the HP extract has been reported to efficiently attenuate oxidative stress, apoptosis and Ca^2+^ entry through modulation of TRPM2 and TRPV1 channels in DRGN of SCI-induced rats (Özdemir et al., 2016). Current results supported results of the reports on HP treatment in the human phagocytic cells and rat DRGNs (Nazıroğlu et al., 2014a,b,c; Özdemir et al., 2016).

TRPC6 channel is belonging to the superfamily of TRP. It was reported that hyperforin caused intracellular Ca^2+^ elevations through TRP canonical 6 (TRPC6) in PC12 cells (Leuner et al., 2007) although other effects of hyperforin are described which might also participate in its pharmacological actions. For example, hyperforin attenuates voltage- and chemical-gated Ca^2+^ conductances in isolated hippocampal neurons and cerebellar Purkinje neurons (Chatterjee et al., 1999; Fisunov et al., 2000). At the cellular level, the hyperforin induced mitochondrial membrane depolarization through releasing zinc and calcium ions from these intracellular organelles (Tu et al., 2010). Contrary, depletion of intracellular Ca^2+^ stores with the SERCA pump inhibitor (thapsigargin) did not affect hyperforin-induced [Ca^2+^]_i_ transients although hyperforin increased Ca^2+^ entry through TRPC6 channel activation in primary hippocampal neurons (Leuner et al., 2013). Decrease of indomethacin-induced Ca^2+^ mobilization, cytotoxicity, apoptosis, and caspase activation in Caco-2 cell line was reported by quercetin as a component of HP (Carrasco-Pozo et al., 2012). Hyperforin also induced Ca^2+^ transients in dissociated primary cultures of embryonic cortical neurons through channels displaying TRPC6-like properties (Tu et al., 2009). Recently it was reported that that hyperforin induces TRPC6-independent hydrogen ion currents in HEK-293 cells, cortical microglia, chromaffin cells, and lipid bilayers (Sell et al., 2014). No association between, hyperforin-induced apoptosis, TRPC6 activation and oxidative stress in neonatal pig glomerular mesangial cell was reported (Soni and Adebiyi, 2016). Contrary, cerebral ischemia-induced rat cortical neuron TRPC6 degradation, oxidative stress and apoptosis were reduced at 24 h of cerebral ischemia by hyperforin treatment (Lin et al., 2013). According to the conflicting results, the mechanisms of hyperforin on TRPC6 are not fully understood and its effect on the channel seems cell specific and different from antioxidant effect on TRPM2 and TRPV1 in sciatic nerve and DRGN.

In summary, our study provided for the first time that apoptotic pathway, overload Ca^2+^ entry, and mitochondrial ROS production through increased activation of TRPM2 and TRPV1 were increased in sciatic nerve and DRGNs of SNI-induced rats. We identified that SNI-induced sensitization of TRPM2 and TRPV1 activity to induce apoptosis and oxidative stress in the neurons was decreased through modulation of the channels by HP treatment. Inhibition of the channels through HP treatment was probably mediated by direct inhibiting ROS to decrease channel gating. Therefore, current results provide that HP acts a neuronal modulator role against ROS-induced apoptosis Ca^2+^ mobilization through inhibition of TRPM2 and TRPV1 channels in sciatic nerve and DRGNs. This finding is of particular significance and may provide an explanation for the SNI-induced neuronal survival and peripheral pain reduce properties of HP. TRPM2 and TRPV1 channels may become an important pharmacological target in the treatment of SNI-induced apoptosis and pain.

## Ethics statement

The study was approved by the Local Experimental Animal Ethical Committee of Suleyman Demirel University (SDU) (Protocol number: HADYEK-07-2015). The study was performed in accordance with the National Institutes of Health Guide for the Care and Use of Laboratory Animals and the European Community's Council Directives (86/609/EEC). All experiments were carried out in accordance with the approved guidelines.

## Author contributions

MN and FU formulated the present hypothesis and MN was responsible for writing the report. FU was responsible for induction of SNI. BÇ was responsible for sciatic nerve, DRGN isolation and cytosolic Ca^2+^ release analyses. Graphical abstract figure was produced by BÇ.

### Conflict of interest statement

The authors declare that the research was conducted in the absence of any commercial or financial relationships that could be construed as a potential conflict of interest.

## Abbreviations

[Ca^2+^]_i_: intracellular free calcium ion
ACA: N-(p-amylcinnamoyl) anthranilic acid
CAPS: capsaicin
CapZ: capsazepine
CPx: cumene hyroperoxide
DHR: dihydrorhodamine
DRGN: dorsal root ganglion neuron
HP: *Hypericum Perforatum*
PARP-1: Poly-ADPR polymerase 1
ROS: reactive oxygen species
SCI: spinal cord injury
SNI: sciatic nerve injury
TRP: transient receptor potential
TRPC6: transient receptor potential canonical 6
TRPM2: transient receptor potential melastatin 2
TRPV1: transient receptor potential vanilloid 1
WC: whole cell.
